# Long-term hyperglycemia aggravates α-synuclein aggregation and dopaminergic neuronal loss in a Parkinson’s disease mouse model

**DOI:** 10.1186/s40035-022-00288-z

**Published:** 2022-03-07

**Authors:** Yi-Qing Lv, Lin Yuan, Yan Sun, Hao-Wen Dou, Ji-Hui Su, Zhi-Pan Hou, Jia-Yi Li, Wen Li

**Affiliations:** 1 Laboratory of Research in Parkinson’s Disease and Related Disorders, Health Sciences Institute, China Medical University, Shenyang, 110122 China; 2 Neural Plasticity and Repair Unit, Department of Experimental Medical Sciences, Lund University, 221 84 Lund, Sweden

**Keywords:** Hyperglycemia, Diabetes mellitus, Parkinson’s disease, Neurodegeneration, Neuroinflammation

## Abstract

**Background:**

Growing evidence suggests an association between Parkinson’s disease (PD) and diabetes mellitus (DM). At the cellular level, long-term elevated levels of glucose have been shown to lead to nigrostriatal degeneration in PD models. However, the underlying mechanism is still unclear. Previously, we have elucidated the potential of type 2 diabetes mellitus (T2DM) in facilitating PD progression, involving aggregation of both alpha-synuclein (α-syn) and islet amyloid polypeptide in the pancreatic and brain tissues. However, due to the complicated effect of insulin resistance on PD onset, the actual mechanism of hyperglycemia-induced dopaminergic degeneration remains unknown.

**Methods:**

We employed the type 1 diabetes mellitus (T1DM) model induced by streptozotocin (STZ) injection in a transgenic mouse line (BAC-α-syn-GFP) overexpressing human α-syn, to investigate the direct effect of elevated blood glucose on nigrostriatal degeneration.

**Results:**

STZ treatment induced more severe pathological alterations in the pancreatic islets and T1DM symptoms in α-syn-overexpressing mice than in wild-type mice, at one month and three months after STZ injections. Behavioral tests evaluating motor performance confirmed the nigrostriatal degeneration. Furthermore, there was a marked decrease in dopaminergic profiles and an increase of α-syn accumulation and Serine 129 (S129) phosphorylation in STZ-treated α-syn mice compared with the vehicle-treated mice. In addition, more severe neuroinflammation was observed in the brains of the STZ-treated α-syn mice.

**Conclusion:**

Our results solidify the potential link between DM and PD, providing insights into how hyperglycemia induces nigrostriatal degeneration and contributes to pathogenic mechanisms in PD.

**Supplementary Information:**

The online version contains supplementary material available at 10.1186/s40035-022-00288-z.

## Background

Parkinson’s disease (PD) is the second most common neurodegenerative disease after Alzheimer’s disease in humans, presenting mainly with motor discoordination accompanied by various non-motor symptoms [1]. Progressive loss of dopaminergic neurons in the substantia nigra (SN) pars compacta (SNpc) and the formation of cytoplasmic inclusions containing misfolded α-synuclein (α-syn), called Lewy bodies (LBs), are two major neuropathological hallmarks of PD [2, 3]. The etiology of PD is not yet clear, with most of the  cases being sporadic, associated with multiple environmental risk factors [4]. Recent studies have revealed a potential relationship between PD and metabolic diseases, such as diabetes mellitus (DM) [5]. DM, characterized by impaired glucose metabolism and subsequent hyperglycemia [6], can be divided into type 1 diabetes mellitus (T1DM) with insufficient insulin secretion due to progressive destruction of pancreatic β cells and type 2 diabetes mellitus (T2DM) with the lack of appropriate insulin response [7–11].

PD is associated with DM specifically in epidemiology, etiology and pathogenesis. First, epidemiologically, DM is present as a risk factor for PD [5, 12]. The elevated risk of developing cognitive abnormalities in individuals with impaired glucose metabolism has been well documented [13, 14]. Multiple studies have indicated that patients with preexisting DM have an increased incidence of parkinsonian symptoms [15–19]. Second, PD and DM share potential contributing factors and have overlapping pathology. Insulin resistance, a common pathogenesis in T2DM, has been shown to contribute to the onset of α-syn pathology in neurons and oligodendrocytes [20]. Some diabetic patients also exhibit pathologies related to striatal dopaminergic dysfunction [10, 21]. Phosphorylated α-syn inclusions have been found in pancreatic β cells of T2DM subjects, indicating the existence of PD-related peripheral pathology in DM [22].

At the mechanistic level, however, the link between PD and DM is not yet clear [23–25]. Our previous study has suggested a possible role of protein co-aggregation during the interplay of the two diseases in a non-human primate model [26]. However, considering the influence of insulin resistance in amyloid protein aggregation [27], the mechanisms of hyperglycemia-induced neurodegeneration remain obscure. Of all the shared potential mechanisms of DM and PD, neuroinflammation is of particular importance [28]. Elevated glucose levels can mediate oxidative stress and peripheral inflammation [29–31], which may subsequently induce diffusion of cytokines into the brain [32, 33], and therefore activate microglia and contribute to the onset of PD [20, 34]. Therefore, the hyperglycemia-induced neuroinflammation may be a mechanistic link from DM to PD.

In this study, we set out to study the effect of hyperglycemia on the nigrostriatal pathway using the streptozotocin (STZ)-induced T1DM model with either wild-type (WT) or α-syn transgenic mice (BAC-α-syn-GFP) background. After characterizing the T1DM phenotypes, we investigated PD-related motor dysfunction, dopaminergic neuronal and terminal loss, and α-syn aggregation in these animals. Moreover, we investigated the neuroinflammation status in the nigrostriatal systems of STZ-injected α-syn mice, exploring the potential mechanistic link between hyperglycemia and PD-related alterations.

## Materials and methods

### Animal grouping

α-Syn-overexpressing mice (BAC-α-syn-GFP) [35] and WT (C57BL/6 J) mice (Huafukang Company, Beijing, China)  at 11–12 weeks were used to generate the STZ-induced diabetic model. The BAC-α-syn-GFP mouse model was generated in our lab and has been described previously [35]. Briefly, the transgenic mice express human WT full-length α-syn fused with green fluorescent protein (GFP) under the mouse α-syn gene promoter, generated by pronuclear inoculation of bacterial artificial chromosome (BAC) to C57BL/6 J background. Mice were maintained at 25 °C with food and water available ad libitum*.*

Both the BAC-α-syn-GFP mice and the WT mice were randomly allocated to sodium citrate buffer treatment (α-syn + vehicle and WT + vehicle) and STZ groups (α-syn + STZ and WT + STZ). Mice were sacrificed at one month and three months after injections. The number of mice per group is listed in Additional file 1: Table S1. All experimental procedures were approved by the Research Ethics Committee of China Medical University and performed according to the international guidelines.

### Generation and characterization of STZ-induced T1DM mice

**Fig. 1:**
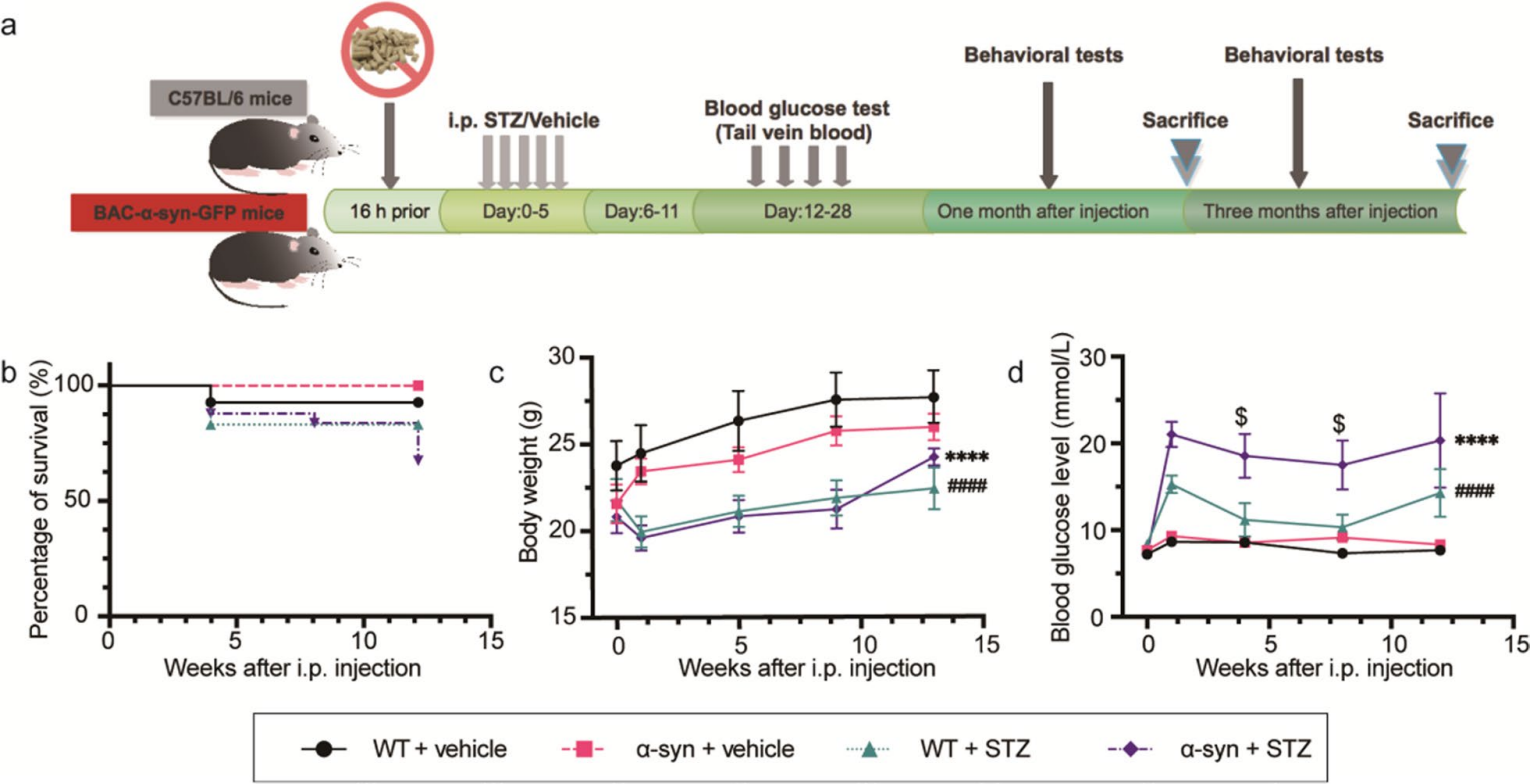
STZ treatment reduces the body weight and induces more severe hyperglycemia in α-syn-overexpressing mice. (**a**) Schematic of the experimental design. Both WT and BAC-α-syn-GFP mice were injected (i.p.) with STZ or vehicle. Body weight and blood glucose level were routinely monitored throughout the experiment. The mice were subjected to behavioral tests at one month and three months after injections. (**b**) Percentage of survival at 12 weeks after STZ i.p. injections. (**c**, **d**) Body weight and blood glucose alterations after STZ i.p. injections. The number of mice per group was provided in Additional file 1: Table S1. ****P < 0.0001 α-syn + STZ *versus* α-syn + vehicle, ^####^*P* < 0.0001 WT + STZ* versus* WT + vehicle, ^$^*P* < 0.05 WT + STZ *versus* α-syn + STZ

Low-dose (70 mg/kg) STZ (Sigma-Aldrich, Shanghai, China, #S0130) or control buffer was intraperitoneally (i.p.) injected daily for 5 consecutive days [36]. After 16-h food deprivation, tail vein blood was collected for glucose determination using an electronic scale (YuWell, China) 12 h after fasting. Body weight and blood glucose were measured 0, 1, 4, and 7 weeks  as well as 3 months after the last injection (Fig. 1a). Blood glucose value > 150 mg/dl (8.3 mmol/l) was considered as successful induction of diabetes.

### Behavioral tests

Pole test was used to assess the motor coordination and balance of mice, and open field test (OFT) was used to assess the spontaneous activity of mice, as previously described [37, 38]. In the pole test, a steel pipe with length of 55 cm and diameter of 1 cm was tightly wrapped in white antiskid gauze and fixed on plastic foam base. A spherical protrusion with diameter of 2 cm was placed on top of the pipe as attachment point for mice. During the experiment, a mouse was put on the spherical protruding point with head upward. The time of the mouse to turn from upwards to a downwards position (T-turn) and the total time (T-total) to climb down the pole were recorded.

OFT was used to assess anxiety and spontaneous activity of mice. Briefly, mice were placed in the test room for an hour to adapt to the surroundings. Then the mice were put in the center of an open field (90-cm long, 90-cm wide, and 40-cm high, divided into 9 squares) and monitored for 15 min by an overhead digital camera connected to an automated video-tracking system (Harvard Apparatus, Massachusetts, USA, model: SMART® v 3.0). The mean speed and times in the total zone, the total distance travelled, the distance travelled in the central zone and the distance travelled in the periphery zone were recorded.

### Immunohistochemistry and immunofluorescence

Coronal sections of 30-µm thick were cut from 4% paraformaldehyde-fixed brain and paraffin-embedded pancreatic tissues, then sectioned into 5-μm-thick slices encompassing the islets, using a rotary microtome (RM2016; Leica Microsystems GmbH, Beijing, China). Fresh brain tissues were also collected and frozen for protein analyses. For diaminobenzidine (DAB) (Vector Laboratories, Pennsylvania, USA, # SK-4100) staining, brain sections and deparaffinized pancreatic slides were subjected to antigen retrieval in citrate acid buffer (pH 6.0) at 80 °C for 30 min, quenched with peroxidase solution (3% H_2_O_2_ in 10% methanol) for 15 min, and blocked in 5% normal goat or horse serum for 1 h. Primary antibody incubation (Additional file 1: Table S2) was performed at 4 °C overnight followed by biotinylated secondary antibody incubation at room temperature for 1 h. After avidin–biotin complex (ABC) (Vector Laboratories, Pennsylvania, USA, # VEPK-6100) and DAB incubations, sections were mounted with neutral balsam for microscopic analyses. Fluorescence-stained sections were mounted with an anti-fading medium for confocal analysis (Leica TCS SP8, Germany).

### Western blot

Mouse brain SN tissues were homogenized by ultrasonic crushing on ice. After SDS-PAGE, polyvinylidene difluoride membranes (Millipore, Beijing, China) were blocked in 5% skimmed milk (Millipore, Beijing, China) in TBS with 0.05% Tween-20. Primary antibody incubation was performed overnight at 4 °C followed by incubation with horseradish peroxidase (HRP)-conjugated antibody at room temperature for 1 h. ECL kits (Tanon, Shanghai, China, #180–5001) were used for chemiluminescence imaging  analysis (Tanon, Shanghai, China, #5500). For detection of soluble and insoluble fractions of α-syn, 1% Triton X-100 buffer was used to dissolve the homogenized tissues. Lysates were centrifuged at 20,000*g* for 30 min at 4 °C to separate the insoluble material, with the supernatant referred to as the Triton-soluble fraction. Both soluble and insoluble proteins were then subjected to SDS-PAGE. Whole membranes were incubated with α-syn antibodies.

### Stereology

Unbiased quantification of the total number of tyrosine hydroxylase (TH)- and serine 129 phosphorylated α-syn (pS129-α-syn)-positive neurons in the SN was performed according to the optical fractionator principle [39], using a stereological system including 2-dimensional anatomical mapping and a cell quantification program (Stereo Investigator; MBF Bioscience, Williston, ND) coupled to a color brightfield microscope (Nikon, Shanghai, China, M570E). A full series of sections (6 sections) per mouse was used for counting. The virtual outlines of the right and left SN were drawn in a manner consistent with the description in a previous article [39]. Stereo Investigator’s Virtual Slice module was used to draw interesting counting regions at low magnification. Starting at a random field, the number of stained neurons was counted under high magnification (100 × objective).

The total numbers of TH-positive and pS129-α-syn-positive neurons in the SN were calculated according to the following equation:

$$N = { 1}/SSF \times { 1}/ASF \times { 1}/TSF \times \sum {\text{Q}}^{ - }$$

*N* stands for the number of target neurons, SSF represents the section sampling fraction, ASF means the area of sampling fraction and TSF is the thickness of sampling fraction. TSF was calculated as dissector height/mean thickness and ƩQ^−^ is the number of neurons counted. For this investigation, SSF = 6 and ASF = 60%. The Gundersen coefficient of error (CE) was used to determine the sufficiency of the samples (CE < 0.1).

The counting point of a neuron was where the nuclear membrane was in sharp relief while focusing up and down in the z-axis, when the estimated center of the nuclei was within the counting frame and contour lines simultaneously, without touching the counting-frame line or contour line in the x/y-axis.

### Quantification

The area of islet β-cell area was quantified with insulin staining. Five different islet structures in at least three mice in each group were included. The area measurements of the whole islet and insulin-positive areas were determined by the Image J software (version 1.34.3.67; National Institutes of Health, Bethesda, MD). The average percentage of insulin-positive area in the whole islet was employed to evaluate the damage of pancreatic tissues.

The number of microglia was counted using the Manual Cell Counting and Marking function of the Image J software. Morphological grading of microglia was performed using a standard protocol published previously [40]. Co-localization between the major histocompatibility complex Class II (MHC class II) and ionized calcium binding adapter molecule 1 (Iba-1) in the striatum was quantified using the Pearson’s Correction with the Fiji plugin Coloc 2 [41].

### Statistical analysis

Statistical analyses were performed with the GraphPad Prism 8 software. Three-way analysis of variance (ANOVA) and two-way ANOVA with Dunnett’s multiple comparisons and Tukey’s multiple comparisons were employed. All values are presented as the mean ± standard error of the mean (SEM). *P* values are described in figure legends.

## Results

### Mortality and body weight loss in STZ-injected mice

During routine monitoring of body weight and blood glucose level, 20% (8 of 40) of the BAC-α-syn-GFP mice died following STZ treatment, among which 3 died within the first month after injection, and 5 died during the following two months. In the WT + STZ group, 3 mice died in the first month following injections, possibly due to an acute toxic effect of STZ; and no death in the following month (Fig. 1b). All STZ-injected mice developed a significant loss of body weight during the first two weeks  after injections. Although there was a trend of body weight increase in all groups during the following months, the body weights of the mice with i.p. STZ (WT + STZ, α-syn + STZ) were significantly lower than those with vehicle injection (WT + vehicle, α-syn + vehicle) (Fig. 1c). The significantly lower body weights in the STZ-injected groups suggest a disrupted metabolic status.

### STZ treatment induces more severe hyperglycemia in α-syn-overexpressing mice compared to WT mice

STZ treatment increased the blood glucose levels in both WT and BAC-α-syn-GFP mice from the first week after injections, and the levels remained at a high level in the following weeks (Fig. 1d). In the α-syn + STZ group, the blood glucose levels ranged from 16.3 to 21.0 mmol/l, while in the WT + STZ group the blood glucose levels were from 10.4 to 15.28 mmol/l. The blood glucose levels of the α-syn + STZ group were significantly higher than that in the WT + STZ group throughout the experimental period (three months) (Fig. 1d), indicating the successful induction of the T1DM diabetic mouse model and more severe hyperglycemia in the α-syn-overexpressing mice than in the WT mice. In contrast, the blood glucose levels remained stable in both vehicle-treated groups (WT + vehicle mice, 7.3–8.7 mmol/l; α-syn + vehicle mice, 8.3–9.3 mmol/l).

### STZ treatment exacerbates structural damage of islets in α-syn-overexpressing mice than in WT mice

**Fig. 2:**
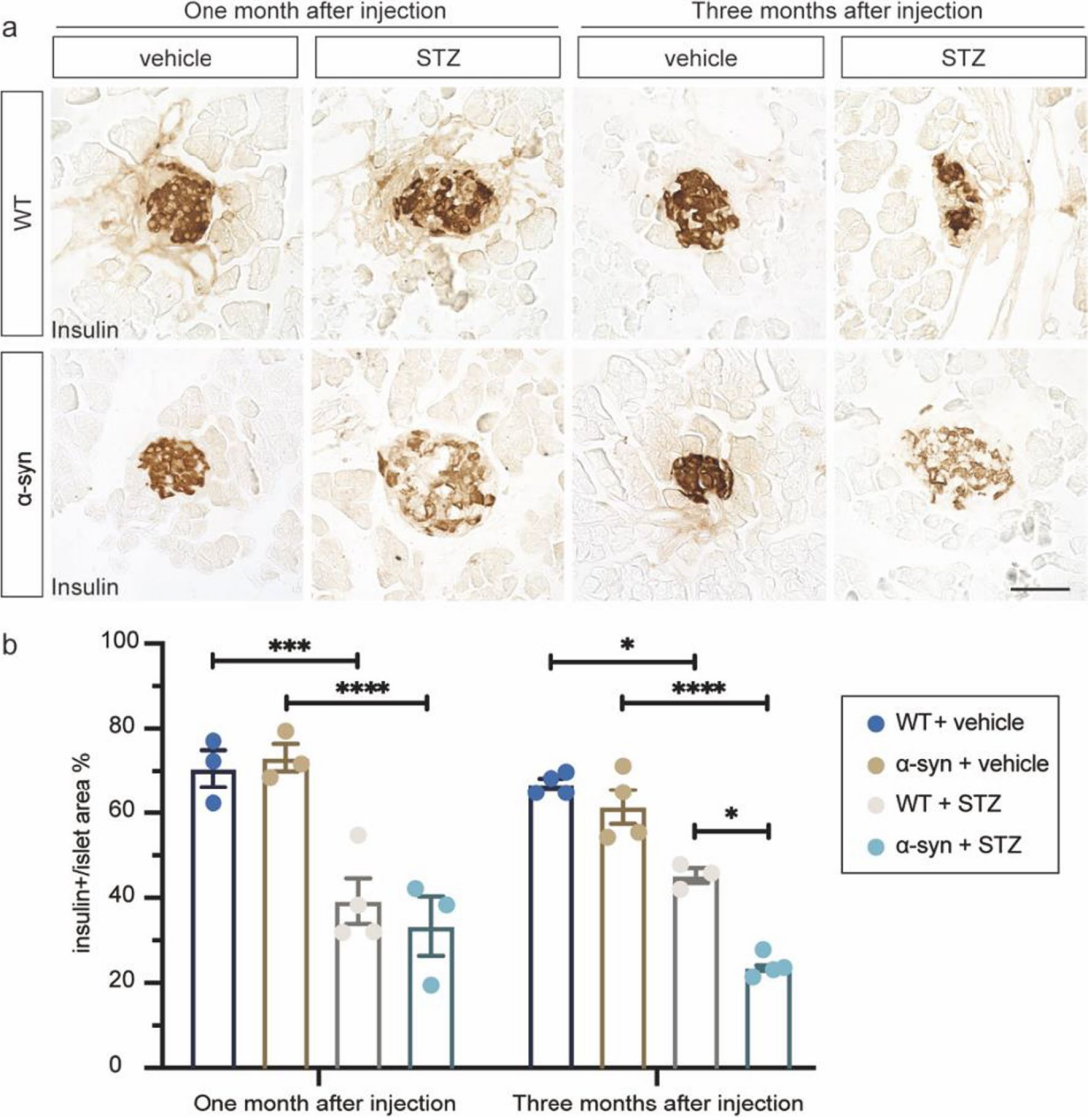
STZ treatment exacerbates structural damage of islets in α-syn-overexpressing mice compared to WT. (**a**) Representative microscopic images of immunohistochemical staining with antibody against insulin at one and three months after injections, in the pancreatic islets of WT + vehicle, WT + STZ, α-syn + vehicle and α-syn + STZ groups. (**b**) Quantification of average percentage of islet β-cell area to the total islet area in pancreas of WT and BAC-α-syn-GFP mice with i.p. injections of STZ or vehicle (*n* = 3 to 4 mice/group). Scale bar, 50 µm. *P < 0.05, ***P < 0.001, ****P < 0.0001

The STZ-injected mice showed a significant decrease in the insulin-positive profile in pancreas. The average percentage of insulin-positive area to the total islet area decreased by 31.42% and 21.7% in WT mice and 39.94% and 37.89% in BAC-α-syn-GFP mice, at one and three months after injections, respectively (Fig. 2a, b). Neither WT nor BAC-α-syn-GFP mice with vehicle injection showed decreased insulin immunoreactivity. Notably, the percentage of insulin-positive area to the islet area in pancreases of the α-syn + STZ mice was significantly lower than that of the WT + STZ mice by 21.65% at three months post-injection (Fig. 2b), indicating a more severe pancreatic β cell loss in the α-syn mice. The pathological alterations in the pancreases suggest that the STZ-treated mice exhibited features reminiscent of T1DM [42], and more importantly, α-syn exacerbated the loss of islet β-cells.

### Long-term hyperglycemia aggravates motor deficits in α-syn-overexpressing mice

**Fig. 3:**
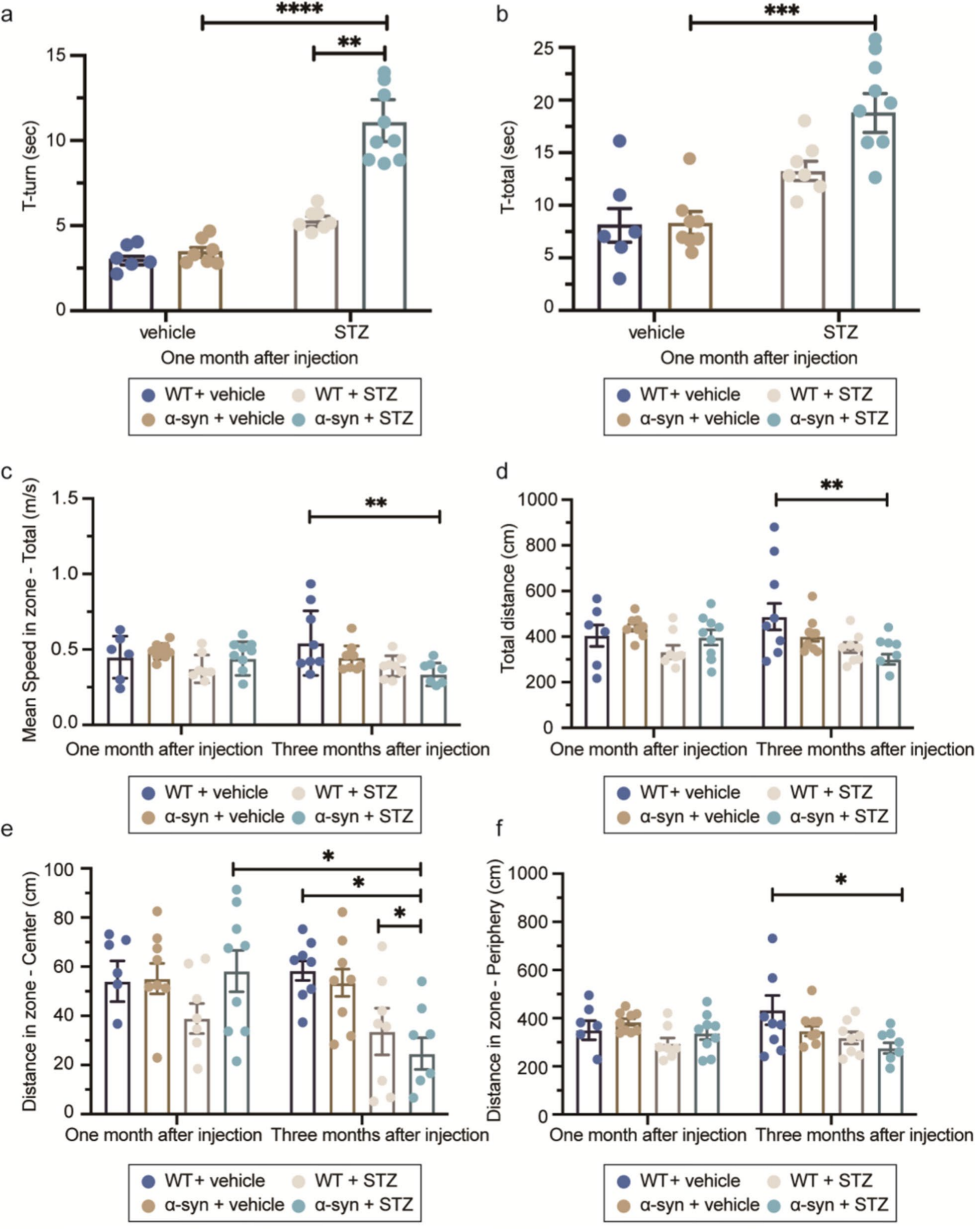
Long-term hyperglycemia aggravates motor deficits in α-syn-overexpressing mice. Motor performance at one and three months after injections in the pole test (**a**, **b**) and open field test (**c**–**f**). T-turn (**a**) and T-total (**b**) increased in the STZ-treated α-syn compared to vehicle. (**c**–**f**) In the open field test that assesses the spontaneous activity of mice, the mean speed in total zone (**c**), the total distance (**d**), the distance in the central zone (**e**) and the distance in the peripheral zone (**f**) differed between the α-syn + STZ and WT + vehicle groups. At least three mice were included in each group. The number of mice per group was provided in Additional file 1: Table S1. *P < 0.05, ***P < 0.001, ****P < 0.0001

The pole test and open field test were performed to assess the motor performance and the functional degeneration in the nigrostriatal pathway. In the pole test, both T-turn and T-total were significantly longer in the α-syn + STZ group than in the WT + STZ and vehicle-treated groups at one month after injection, suggesting that hyperglycemia aggravates the motor deficits in the α-syn mice compared to the vehicle (Fig. 3a, b). At three months post-injection, most of the mice in the α-syn + STZ group were too weak to climb the pole, therefore T-turn and T-total could not be recorded for this group.

In the open field test, at one month after injections, the BAC-α-syn-GFP mice exhibited no significant alterations in spontaneous movement, regardless of the STZ treatment. However, the mean movement speed of the α-syn + STZ mice was noticeably slower than that of the WT + vehicle mice at three months after injections (Fig. 3c). Meanwhile, the total distance travelled (including the distance in the center and in the periphery) by the α-syn + STZ mice was reduced compared to the WT + vehicle mice (Fig. 3d–f). Furthermore, the α-syn + STZ mice travelled a shorter distance than the WT + STZ mice in the central area of the open field at three months after injections (Fig. 3e). The earlier presence of motor deficits in α-syn + STZ compared to α-syn + vehicle mice indicates that hyperglycemia increases the susceptibility of BAC-α-syn-GFP mice to motor impairments (more significant at three months after injections), which might be attributed to dopaminergic damage.

### Long-term hyperglycemia induces dopaminergic neuronal degeneration in α-syn-overexpressing mice

**Fig. 4:**
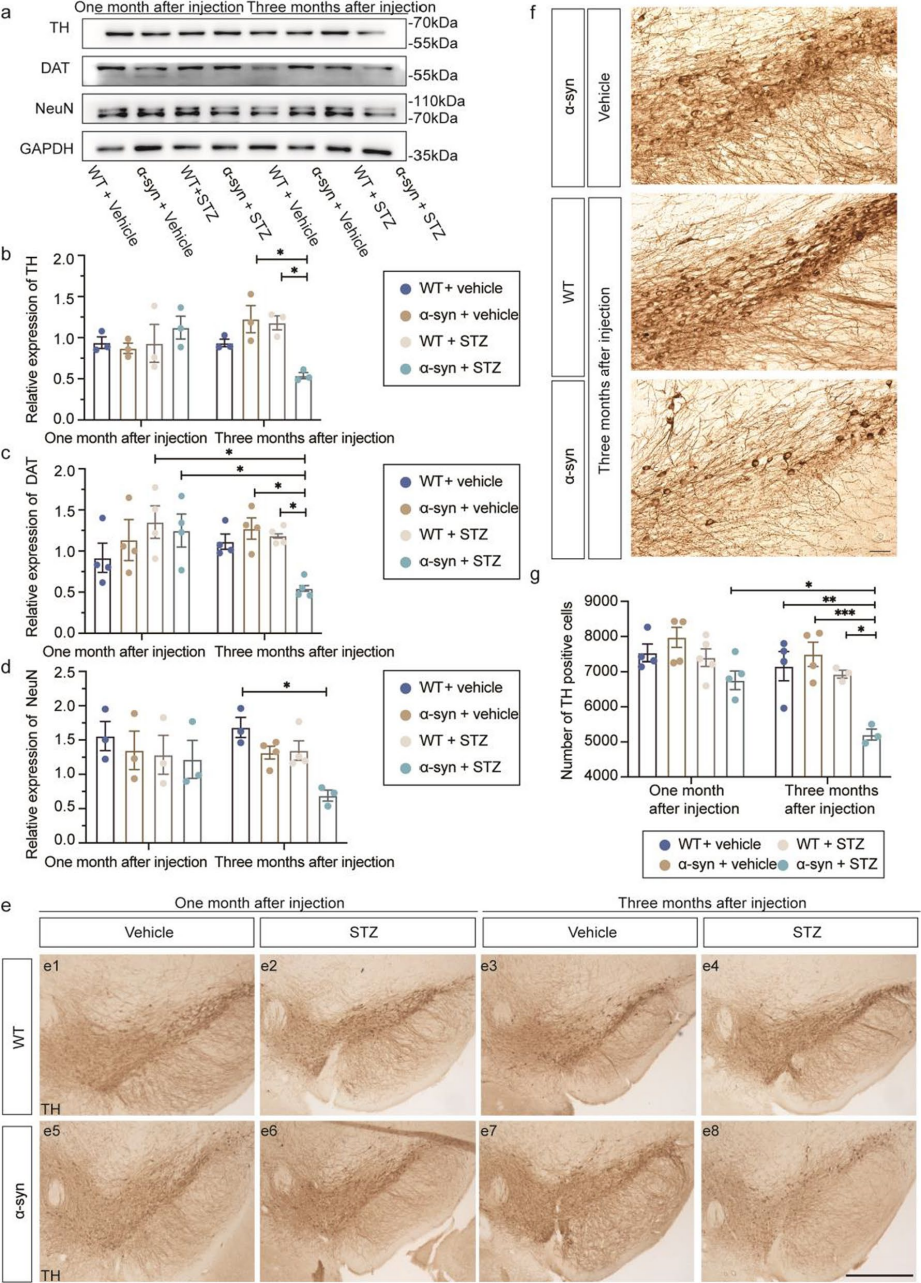
Long-term hyperglycemia causes degeneration of dopaminergic neuronal profile in α-syn-overexpressing mice. (**a**) Western blot analyses of TH, DAT and NeuN protein levels in mouse SNpc. (**b**–**d**) Quantification of TH, DAT and NeuN protein levels (*n* = 3 to 4 mice/group). (**e**) Representative coronal mesencephalic sections with immunohistochemical staining of TH in the SN. (**f**) High-magnification images corresponding to the SNpc area indicated in (**e4**), (**e7**), and (**e8**). (**g**) Quantitative analyses of TH-positive neurons determined by stereology in the midbrain region. WT + vehicle (*n* = 4/4 mice at one/three months after injections, respectively); WT + STZ (*n* = 5/3 mice at one/three months after injections, respectively); α-syn + vehicle (*n* = 4/4 mice at one/three months after injections, respectively); α-syn + STZ (*n* = 4/3 mice at one/three months after injections, respectively). Scale bars, 50 µm. *P < 0.05, **P < 0.01, ***P < 0.001

To investigate whether hyperglycemia has any pathological impact on the nigrostriatal dopaminergic system, we examined the expression of dopaminergic neuronal markers, TH and dopamine transporter (DAT), by Western blotting. There were significant reductions of TH and DAT in the SN of α-syn + STZ mice at three months post-injections, compared to the WT + STZ and α-syn + vehicle groups, suggesting degeneration of dopaminergic neurons (Fig. 4a–c). No difference was observed among groups at one month after injections (Fig. 4a–c). These results were further confirmed by immunohistochemical and stereological analyses, focusing on discrete dopaminergic neurons in the ventral mesencephalon, including the SNpc and the ventral tegmental area (Additional file 1: Fig. S1, Fig. 4e). Mice of the α-syn + STZ group displayed a significant loss of dopaminergic neurons in the SNpc compared to WT + STZ and α-syn + vehicle groups at three months after injections (Fig. 4e–g). Stereological analyses also showed time-dependent neurodegeneration in the α-syn + STZ group by comparison between one month and three months post-injections (Fig. 4g). Furthermore, consistent with the SN, in the striatum, decreased expression levels of TH and DAT were also observed in the STZ-treated α-syn mice at three months after injection (Additional file 1: Fig. S2), indicating that the long-term hyperglycemia causes damage to both dopaminergic neurons and terminals in α-syn mice. The expression levels of the neuronal marker NeuN showed a decrease to a similar extent as that observed for TH, indicating that the neuronal profile change is mainly attributed to the dopaminergic profile in the SN (Fig. 4d, Additional file 1: Fig. S3).

### Hyperglycemia increases α-syn phosphorylation and aggregation in the SN

**Fig. 5:**
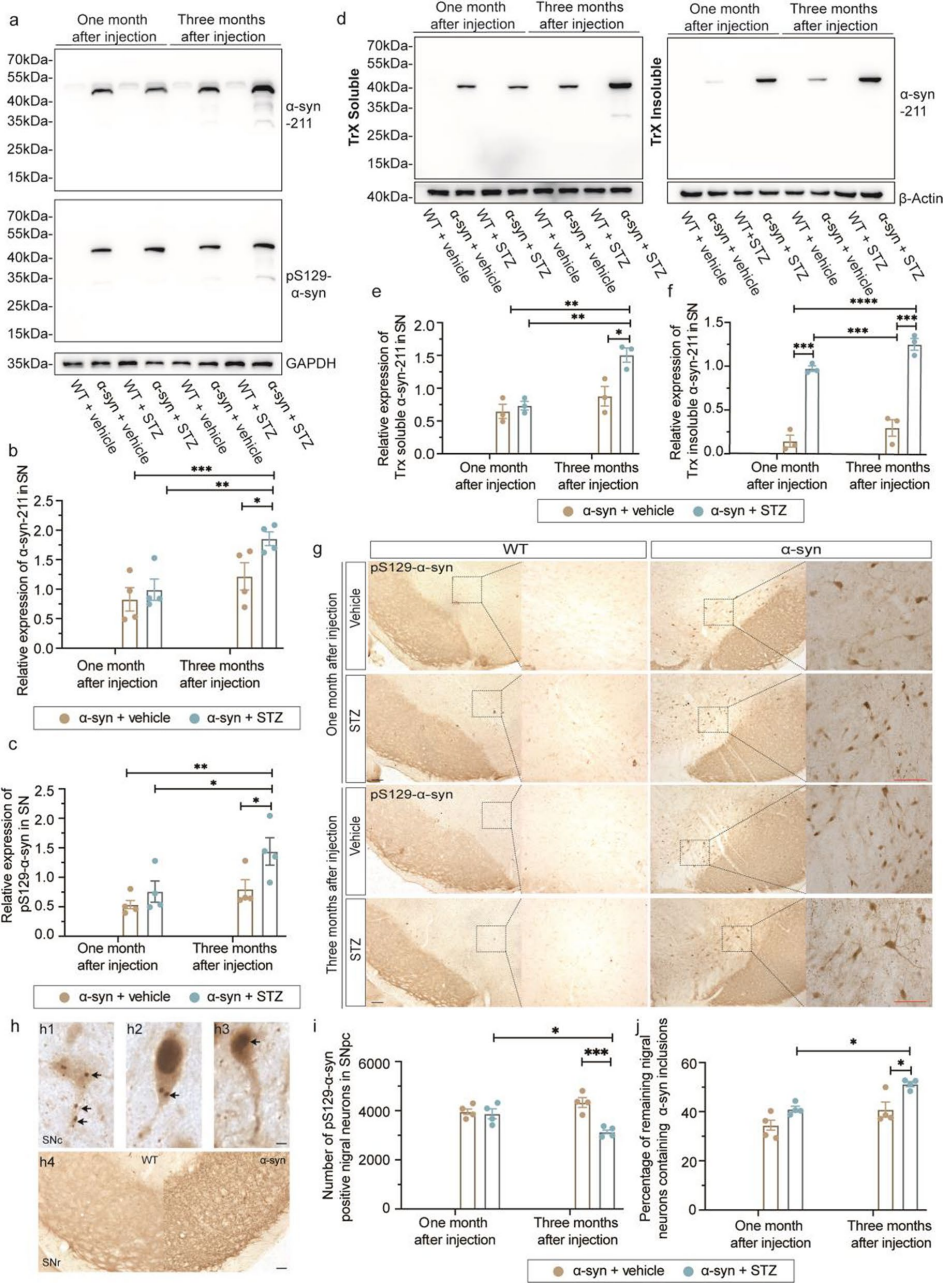
Hyperglycemia increases α-syn aggregation and phosphorylation in the nigrostriatal system in α-syn-overexpressing mice. (**a**) Western blot analyses of α-syn and phosphorylated α-syn (pS129) levels in mouse SN. (**b**, **c**) Quantification of α-syn and pS129-α-syn protein levels (*n* = 3 to 4 mice/group). (**d**) Western blot analyses of Triton-soluble α-syn (Trx soluble) and Triton-insoluble α-syn (Trx insoluble) in mouse SN. (**e**–**f**) Quantification of relative expression of Trx-soluble and Trx-insoluble α-syn by intensity measurement (*n* = 3 per group). (**g**) Representative immunohistochemical images of pS129-α-syn in the SN of WT and BAC-α-syn-GFP mice at one and three months after injections. Images in the right column are higher magnifications of boxed areas in the left column. (**h**) Representative images of two different types of pS129-α-syn-positive nigral neurons. **h1** and **h2** show neurons containing pS129-positive inclusions, while **h3** shows a neuron with diffused staining. **h4** shows the pS129 staining in SN reticulata, where significant positive profile is present in BAC mice (right panel). (**i, j**) Stereological quantification of pS129-α-syn-positive nigral neurons in the SNpc (**i**) and the proportion of remaining nigral neurons containing α-syn inclusions (**j**). WT + vehicle (*n* = 4/4 mice at one/three months after injections, respectively); WT + STZ (*n* = 4/3 mice at one/three months after injections, respectively); α-syn + vehicle (*n* = 4/4 mice at one/three months after injections, respectively); α-syn + STZ (*n* = 4/4 mice at one/three months after injections, respectively). Scale bars: (**g**) left column, 200 µm; right column, 50 µm; (**h**) **h1**–**h3**, 10 µm; **h4**, 50 µm. *P < 0.05, **P < 0.01, ***P < 0.001, ****P < 0.0001

As a consequence of α-syn overexpression, the BAC-α-syn-GFP mice develop α-syn phosphorylation and aggregation in an age-dependent manner [35]. To determine whether hyperglycemia could worsen the α-syn pathology, we studied α-syn expression and phosphorylation in the mouse brains. Three months after STZ induction, human α-syn expression and phosphorylation increased significantly along with hyperglycemia (Fig. 5a–c), suggesting a link between hyperglycemia and the severity of α-syn pathology. We further examined the characteristics of SN α-syn by analyzing Triton-X100 solubility. The STZ treatment increased the insoluble fraction of α-syn both at one month and at three months, whereas the soluble fraction only increased at three months, suggesting that the aggregated status of nigral α-syn  is enhanced by hyperglycemia (Fig. 5d–f).

Immunohistochemical analyses also revealed signals of phosphorylated α-syn in the BAC-α-syn-GFP SN, but not in the WT mice (Fig. 5g). Notably, during stereological quantification, we observed two profiles of pS129-α-syn staining, one with diffused pS129-α-syn immunoreactivity in the cytoplasm without obvious inclusions (Fig. 5h3), and the other containing pS129-α-syn-positive deposits (Fig. 5h1-2). Three months after STZ injections, the number of cells stained with pS129-α-syn decreased, due to the loss of DA neurons in the SN (Fig. 5i). However, the proportion of remaining neurons containing pS129-α-syn-positive inclusions increased significantly (Fig. 5j) in the SNpc, suggesting an overwhelming α-syn pathology in the residual dopaminergic neurons in the SN. We also performed human α-syn staining in the pancreatic islets and found increased α-syn deposition in the BAC-α-syn-GFP compared to WT mice, with or without STZ treatments (Additional file 1: Fig. S4). This indicated that STZ treatment did not enhance accumulation of α-syn in the pancreases; however, the α-syn overexpression did deposit α-syn pathology in the peripheral tissue.

In summary, the protein analysis and stereological quantification indicated that hyperglycemia exacerbates α-syn phosphorylation and aggregation.

### Hyperglycemia induces severe neuroinflammation in α-syn-overexpressing mice

To investigate the potential mechanism contributing to the pathological interaction between DM and PD, we examined microglial (Iba-1) and astroglial (glial fibrillary acidic protein, GFAP) activation.

**Fig. 6:**
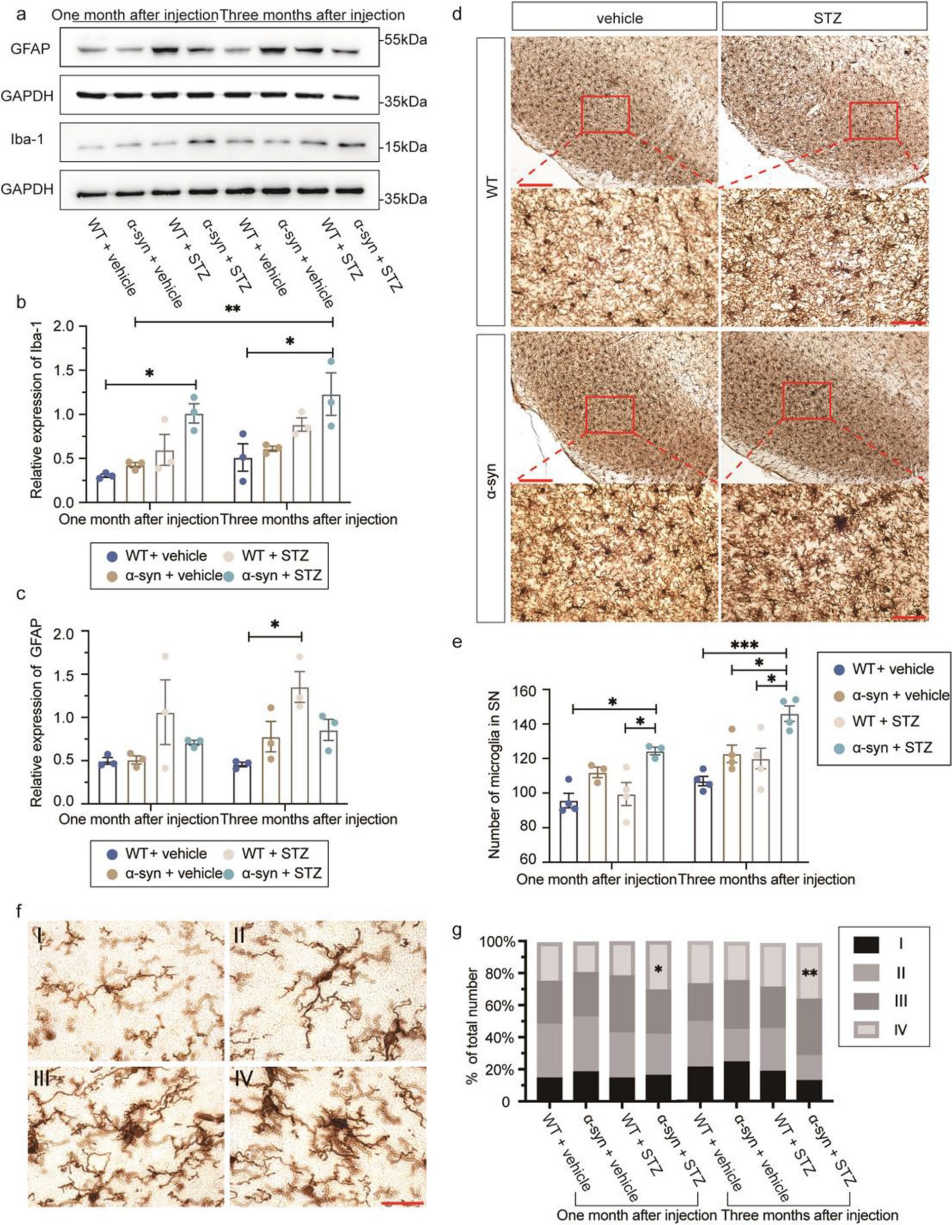
Hyperglycemia promotes neuroinflammation in BAC-α-syn-GFP mice. (**a**) Representative Western blots of Iba-1 and GFAP. (**b**, **c**) Quantification of Iba-1 and GFAP protein levels by intensity measurement (*n* = 3 to 4 mice/group). (**d**) Representative immunohistochemical Iba-1 staining of the SN of mice. (**e**) Number of SN microglia at each time point. (**f**) The morphology of SN microglia at four different activation stages. (**g**) The proportion of four types of microglia based on different morphology. WT + vehicle (*n* = 4/4 mice at one/three months after injection, respectively); WT + STZ (*n* = 4/4 mice at one/three months after injection, respectively); α-syn + vehicle (*n* = 3/4 mice at one/three months after injection, respectively); α-syn + STZ (*n* = 3/4 mice at one/three months after injection, respectively). Scale bars: (**d**), left column, 100 µm; right column, 50 µm; (**f**), 20 µm. *P < 0.05, **P < 0.01 in (**b**, **c**, **e**). *P < 0.05, **P < 0.01 α-syn + STZ versus α-syn + vehicle in stage IV at one and three months after injection (**g**)

By immunoblotting (Fig. 6a), we observed a significant increase in Iba-1 in the SN of α-syn + STZ mice compared to the WT + vehicle mice (Fig. 6b), indicating potential microglial activation resulting from synergic effects of α-syn pathology and STZ treatments. Immunohistochemical analyses showed a significant increase of Iba-1-positive cells in the SN of the α-syn + STZ group at both one and three months after injections, compared to the WT + STZ group (Fig. 6d–e), indicating that hyperglycemia in α-syn mice induces severe neuroinflammation. No difference in Iba-1 immunoreactivity was observed in the SN in the vehicle-injected groups.

Morphological alterations of microglia were further analyzed. The activation of microglia was classified into four stages, including (I) microglia that present small round cell bodies with long thin processes, (II) microglia that have a dense soma and processes remaining thin but longer, (III) microglia that appear in an enlarged and irregular shape, with processes becoming thicker and shorter, and (IV) microglia that become swollen and dark, displaying thickened and branched processes [40] (Fig. 6f). Quantitative analyses of microglia morphological subtypes in the SN region confirmed the hyperglycemia-induced microgliosis in the STZ-injected BAC-α-syn-GFP mice. The stage IV cells, which are deemed to be highly activated microglia with amoeba-like morphology, constituted over 35% and 37% of the total population in the α-syn + STZ group at one and three months after injections, respectively, significantly higher than that of the α-syn + vehicle group (22% and 25%) at the same time points (Fig. 6g). We also observed differences in the expression levels of GFAP in the SN between STZ-injected WT mice and vehicle-injected mice, while in the BAC-α-syn-GFP mice, the activation of astroglia was absent (Fig. 6c).

**Fig. 7:**
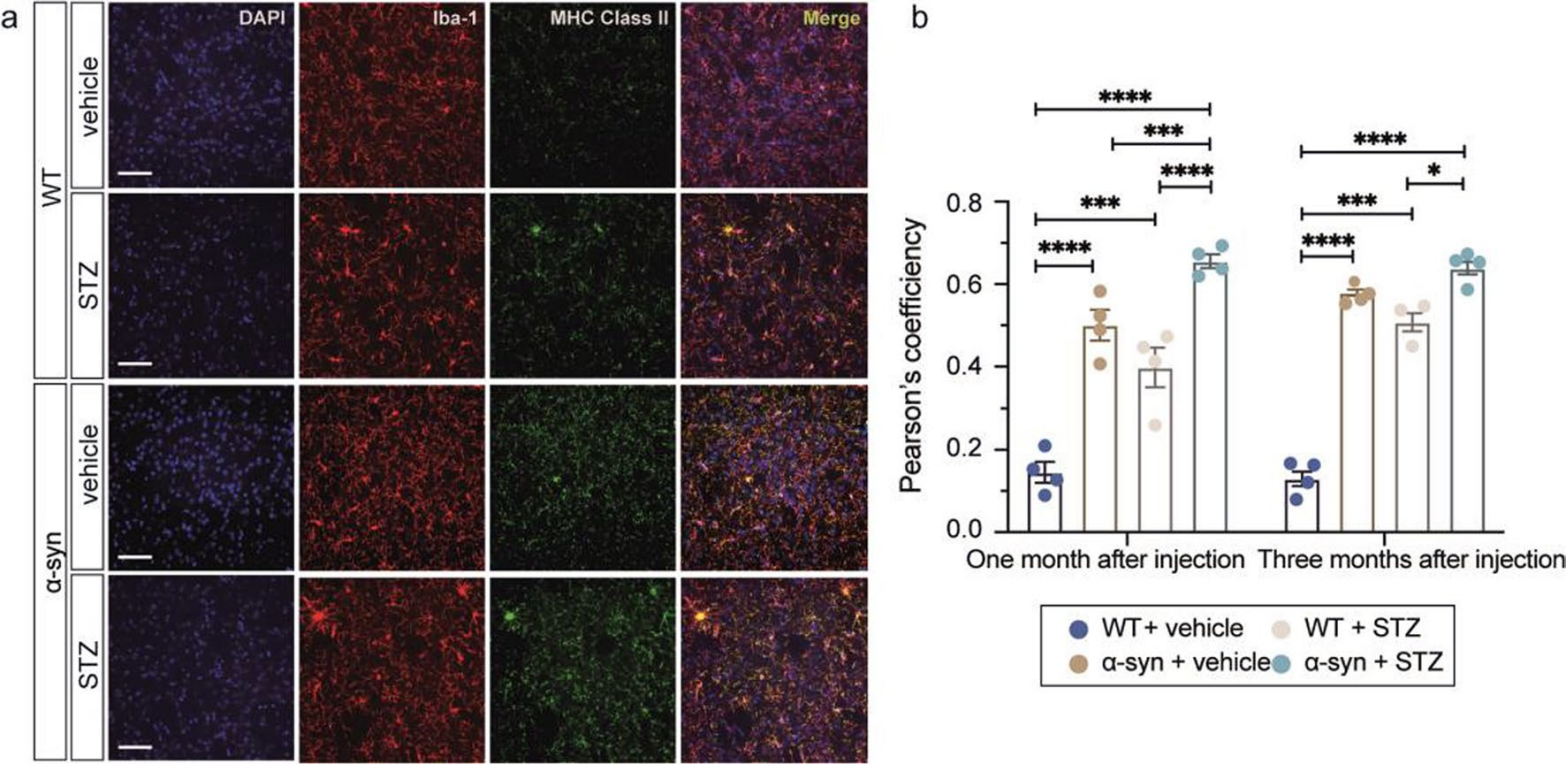
Co-localization of Iba-1 and MHC class II in the striatum of mice. (**a**) Representative images of double-immunofluorescence staining of MHC class II and Iba-1 in the striatum of WT and BAC-α-syn-GFP mice with vehicle or STZ injections. (**b**) Pearson’s coefficient of co-localization between Iba-1 and MHC class II in the striatum (*n* = 4 mice per group). Scale bars, 50 µm. *P < 0.05, ***P < 0.001, ****P < 0.0001

We then examined the functional alterations of activated microglia. Microglia expressing MHC class II in the striatum were significantly increased in the α-syn + STZ group compared to all other groups (Fig. 7a). In addition, the co-localization of MHC class II and Iba-1 was more robust in the striatum in the α-syn + STZ group than in the α-syn + vehicle and WT groups (Fig. 7b, Additional file 1: Fig. S5). The increased expression of MHC class II in microglia indicates an activated status. This result indicates evident neuroinflammation in α-syn-overexpressing mice with hyperglycemia.

## Discussion

Although the exact etiology of PD is still to be uncovered, there exist many risk factors for the onset and progression of the disease. One major risk factor for PD is the impairment of glucose metabolism [25], which may occur years before the first motor symptoms appear, suggesting the particular role of hyperglycemia in facilitating the onset of PD. In this study, we used a hyperglycemic model induced by STZ to cause pancreatic islet damage, to investigate whether and how elevations in blood glucose affect the degeneration process in PD. We employed 3-month-old BAC-α-syn-GFP mice overexpressing α-syn to observe the processes and mechanisms of early-stage PD.

### α-Syn worsens the hyperglycemic phenotype in BAC-α-syn-GFP mice

On comparing the BAC-α-syn-GFP and WT mice with STZ injections, we observed a more severe toxic effect in the BAC mice, based on two sets of data: (1) the consistently higher blood glucose levels and (2) the more severe body weight loss in the BAC mice. Later at three months post injections of STZ, five of the BAC-α-syn-GFP mice died while none of the WT mice died, suggesting a sustained effect of STZ toxicity. There is evidence that the effects of an eight-day treatment with STZ in rodents can be corrected with drug treatments to a non-diabetic status [43, 44]. However, due to the α-syn pathology, our transgenic mice failed to show any remaining effective function of the β cells, where further aggravated T1DM symptoms were observed in the following months. This suggests that the BAC-α-syn-GFP mice are more susceptible to STZ toxicity, which becomes more obvious in the long term.

A question remains to be elucidated on whether α-syn overexpression may worsen the STZ toxicity in the mouse pancreas, and whether α-syn induces any pre-deposited pancreatic pathology. Previously, we have shown that α-syn can deposit in the pancreatic tissue and phosphorylated α-syn can interact with islet amyloid polypeptide  [22]. The expression of α-syn in the pancreatic tissue can be localized on insulin-secreting granules, where overexpression of α-syn inhibits insulin secretion [45]. Mice with α-syn pathology in the pancreas develop reduced glucose-stimulated insulin secretion and insulin resistance at the same time [46]. Therefore, α-syn can possibly aggravate hyperglycemia both at the insulin secretion sites and at the receptor sites [20]. Here, we showed two important results supporting the α-syn toxicity to pancreatic function: (1) α-syn deposited in the pancreatic islets of T1DM diabetic mice to a greater extent compared to non-diabetic mice (Additional file 1: Fig. S4); (2) the insulin content in the BAC mouse pancreas reduced significantly compared to WT mice (Fig. 2a, b). This is consistent with the elevated and all-way higher glucose levels in the STZ-treated BAC mice compared to WT. Moreover, in a current ongoing study in our group, we have found that an α-syn mutant variant is able to cause a severely reduced secretion of insulin (data not shown), which confirms that the α-syn-induced pancreatic pathology is not limited to the specific mouse model we have chosen in this study.

### T2DM *versus* T1DM models to study the direct effect of hyperglycemia on the progression of PD

Despite a few discrepancies, a number of published papers have suggested the association between DM and PD [16, 19, 47], and a majority of these studies focus on the relationship between T2DM and PD. Indeed, T2DM has a much higher incidence than T1DM and it has similarities with PD in aspects of chronic disease progression and dominant ratio in the elderly [10, 48]. Nevertheless, insulin resistance in T2DM affects the regulation of dopaminergic transmission and maintenance of synapses in the central nervous system [49]. The brains of PD patients with T2DM exhibit a process similar to peripheral insulin resistance, suggesting potential common molecular pathways. In animal models, both mice treated with high-fat diets and leptin receptor homozygous deficient (db/db) mice exhibit increased vulnerability to nigrostriatal neurodegeneration [47], again indicating the relationship between T2DM and PD. Our previous studies in T2DM cynomolgus monkeys also indicated that T2DM might facilitate PD onset and progression by interfering with the pathological protein aggregation [26].

However, T2DM itself has complicated etiology and pathology. Both insulin insufficiency and deficiency can occur at the same time [50]. In addition, insulin resistance on its own is challenging to elucidate [49]. Therefore, it would have been rather narrow to attribute the T2DM-induced PD progression solely to hyperglycemia. In addition to hyperglycemia, T2DM is often accompanied by aberrations in lipid metabolism and other related metabolic abnormalities, making the disease background hard to elucidate. Diabetic dyslipidemia is often present among patients with T2DM (72%–85%), while patients with T1DM usually do not have such dyslipidemia (< 10%) [51, 52]. Moreover, such aberrations of lipid metabolism can also be observed in T2DM animal models [26, 53]. Both hyperglycemia and dyslipidemia are associated with PD. Therefore, the contribution of hyperglycemia in a T2DM background to the induction of neurodegeneration remains unclear. In summary, T2DM is possibly more related to PD than T1DM, and a T2DM model may therefore be suitable for studying metabolic disturbance as an early risk factor for PD. However, investigations of a direct effect of hyperglycemia on nigrostriatal degeneration would need a straightforward model harvesting mainly elevated blood glucose levels in a relatively short time.

Compared to T2DM models, the T1DM model with direct damage to insulin secretion certainly possesses a purer background. Although studies focusing on the effect of hyperglycemia caused by T1DM on neurodegenerative diseases are very limited [47, 54], cognitive impairments and structural alterations in the brain are common in adults with T1DM [55], suggesting a close relationship between T1DM and neurodegeneration. In a prospective study, patients with T1DM showed a substantial reduction in limb flexibility and spontaneous movements, associated with brain dysfunction [56]. The direct damage of insulin-producing β-cells makes hyperglycemia a main metabolic pathology of T1DM [57]. The hyperglycemia-induced brain function deficits are an immediate result from the increased glucose levels [58]. Therefore, in this study, we chose the T1DM model to emphasize the direct effect of hyperglycemia on nigral neurons and protein pathology in PD.

### Discrepancy in microglial and astroglial activation

On examining glial activation in the mouse SN, we observed significant microglial activation in multiple aspects, such as cell number, morphology and antigen-presenting capacity. However, astrocyte activation was observed in WT mice but not in BAC-α-syn-GFP mice after STZ treatment. It seems that the additional effects of α-syn in BAC-α-syn-GFP mice were to cause an atrophic effect on reactive astroglia, enhancing the phenotype caused by STZ. In fact, the activation of astroglia and its effect have long been debated in α-syn-related diseases [59]. Minimized astrogliosis in the SN of PD patients has been repored [60, 61], and researchers have raised the possibility of repressing effects of α-syn aggregates on astroglia [62, 63]. Therefore, our findings of astroglia decrease  in the population seem neither unique nor obscure. In the process of neuroinflammation, theoretically the loss of astroglia and the disrupted integrity of astrogliosis could be harmful, as the neuroprotective roles of the cells are no longer present [64]. Therefore, more accurate investigations into the astrocyte status and subtypes in neurodegenerative diseases are still needed.

### Mechanisms of hyperglycemia-induced PD progression

In the present study, we proposed neuroinflammation as the major mechanistic process from hyperglycemia to PD, linking T1DM and PD on the cellular level. In T1DM, how does hyperglycemia cause neuroinflammation? High blood glucose levels can saturate mitochondrial respiration in endothelial cells, astrocytes and pericytes, which in turn promotes reactive oxygen species (ROS) production and oxidative stress [65, 66]. When a high glucose level is maintained for a long time, enhanced ROS and inflammatory cytokines may interrupt the integrity of the blood–brain barrier (BBB) [30]. The integrity of the BBB is essential to maintain the brain environment homeostasis by limiting the pass-through of peripheral immune cells and toxins [67]. Upon penetration by peripheral proinflammatory factors, microglia are activated, and neuroinflammation occurs. Neuroinflammation has been commonly recognized as a crucial feature of PD pathogenesis [68]. Therefore, a chain of events as described above constitute the whole mechanistic process of hyperglycemia-induced neuronal damage. In this study, only neuroinflammation was examined; further research into the exact point(s) of action underlying the cellular changes caused by high levels of blood glucose, such as increased oxidative stress, overproduction of ROS, mitochondrial dysfunction and leakage of the BBB could be very informative.

### Hyperglycemia: a potential risk factor but not a direct initiator in PD pathogenesis

In our study, we did not observe any α-syn aggregation in diabetic WT mice. This is consistent with the behavioral results and the observed TH downregulation. The STZ-induced T1DM model in this study is an acute islet injury model rather than a slowly progressing diabetic model, making it less likely to recapitulate the slow, progressive aggregation of α-syn in WT mice. Hyperglycemia on its own fails to induce the protein pathology of PD. Previous studies have shown that T2DM triggers α-syn accumulation, aggregation and phosphorylation in pancreatic β cells and the brain [22, 24, 26]. Impaired insulin signaling in T2DM causes neuronal insulin resistance in patients, which promotes α-syn accumulation [20, 69]. In the T2DM animal models, the joint effects of multiple metabolic deficiencies, including hyperglycemia and insulin resistance, lead to pathological protein changes. Hyperglycemia alone, at least in the short term, is not able to initiate neurodegeneration. However, hyperglycemia still serves as a risk factor for PD from the perspective of protein pathology. In our PD mice, hyperglycemia can accelerate the already-existing PD pathology. α-Syn accumulation during ageing showed more severe aggregation and phosphorylation after STZ injections than the vehicle groups.

## Conclusion

In conclusion, our findings showed that hyperglycemia aggravates PD progression. STZ-induced α-syn transgenic T1DM mice exhibited significant depletion of dopaminergic profiles, increased α-syn accumulation and phosphorylation in the nigrostriatal system and deficiency in motor performances. In addition, we observed severe neuroinflammation in the α-syn + STZ mice and concluded neuroinflammation as one of the mechanisms contributing to the hyperglycemia-induced nigrostriatal degeneration. Overall, our study bridges metabolic disorders and neurodegeneration from the perspective of cellular mechanism, providing insights into the etiology of PD and age-related metabolic and neuronal disorders.

## Supplementary Information

**Additional file 1:** Figures S1–S5 and Tables S1–S2.

## Data Availability

All data generated or analyzed during this study are included in this published article (and its Additional file 1).

## Abbreviations

α-syn: Alpha-synuclein
ANOVA: Analysis of variance
BAC: Bacterial artificial chromosome
BBB: Blood–brain barrier
CE: Coefficient of error
DAB: Diaminobenzidine
DAT: Dopamine transporter
DM: Diabetes mellitus
GFAP: Glial fibrillary acidic protein
GFP: Green fluorescent protein
HRP: Horseradish peroxidase
Iba-1: Ionized calcium binding adapter molecule 1
i.p.: Intraperitoneal injection
LBs: Lewy bodies
MHC class II: Major histocompatibility complex Class II
OFT: Open field test
PD: Parkinson’s disease
ROS: Reactive oxygen species
SN: Substantia nigra
SNpc: Substantia nigra pars compacta
STZ: Streptozotocin
T1DM: Type 1 diabetes mellitus
T2DM: Type 2 diabetes mellitus
TH: Tyrosine hydroxylase
