# Supplementary material for: Long-term hyperglycemia aggravates α-synuclein aggregation and dopaminergic neuronal loss in a Parkinson’s disease mouse model
Source: Transl Neurodegener. 2022 Mar 7;11:14. doi: 10.1186/s40035-022-00288-z (PMC8900445; doi:10.1186/s40035-022-00288-z)
Supplement: Supplementary file 1 — Additional file 1: Figures S1–S5 and Tables S1–S2. [file 40035_2022_288_MOESM1_ESM.docx]

**Additional file 1**

**
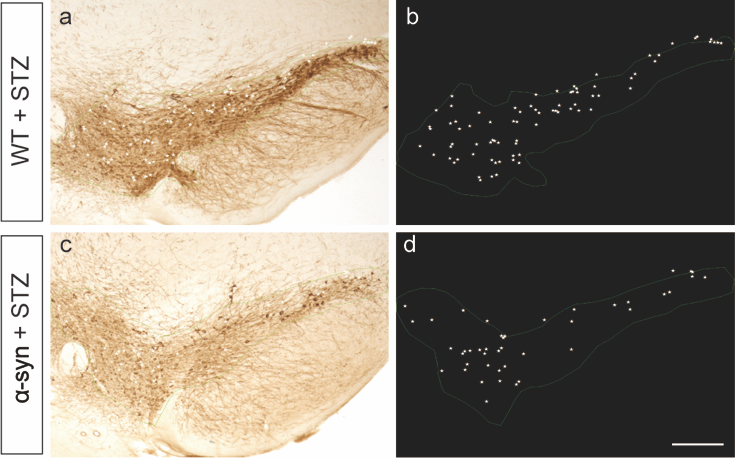
**

**Fig. S1** Contours were drawn around the midbrain sub-region (the SNpc and the VTA) in a manner consistent with the description provided by Baquet *et al*. Image scale bars: 20 µm.


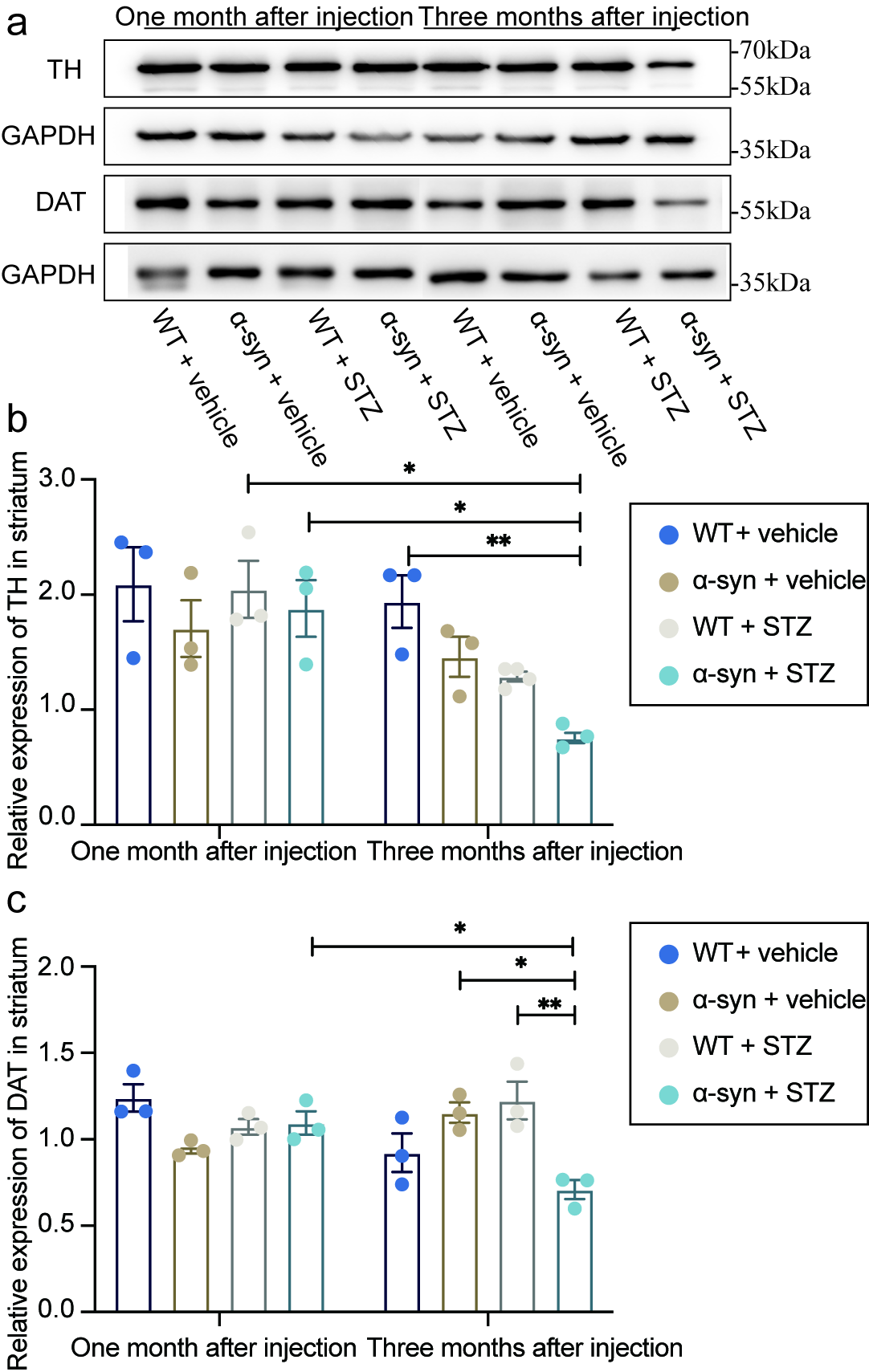


**Fig. S2** (**a**) Representative immunoblot of TH and DAT in the striatum. (**b-c**) Quantification of relative expression of TH and DAT was performed by intensity measurement. (n=3 to 4 mice/group) *P<0.05, **P<0.01.


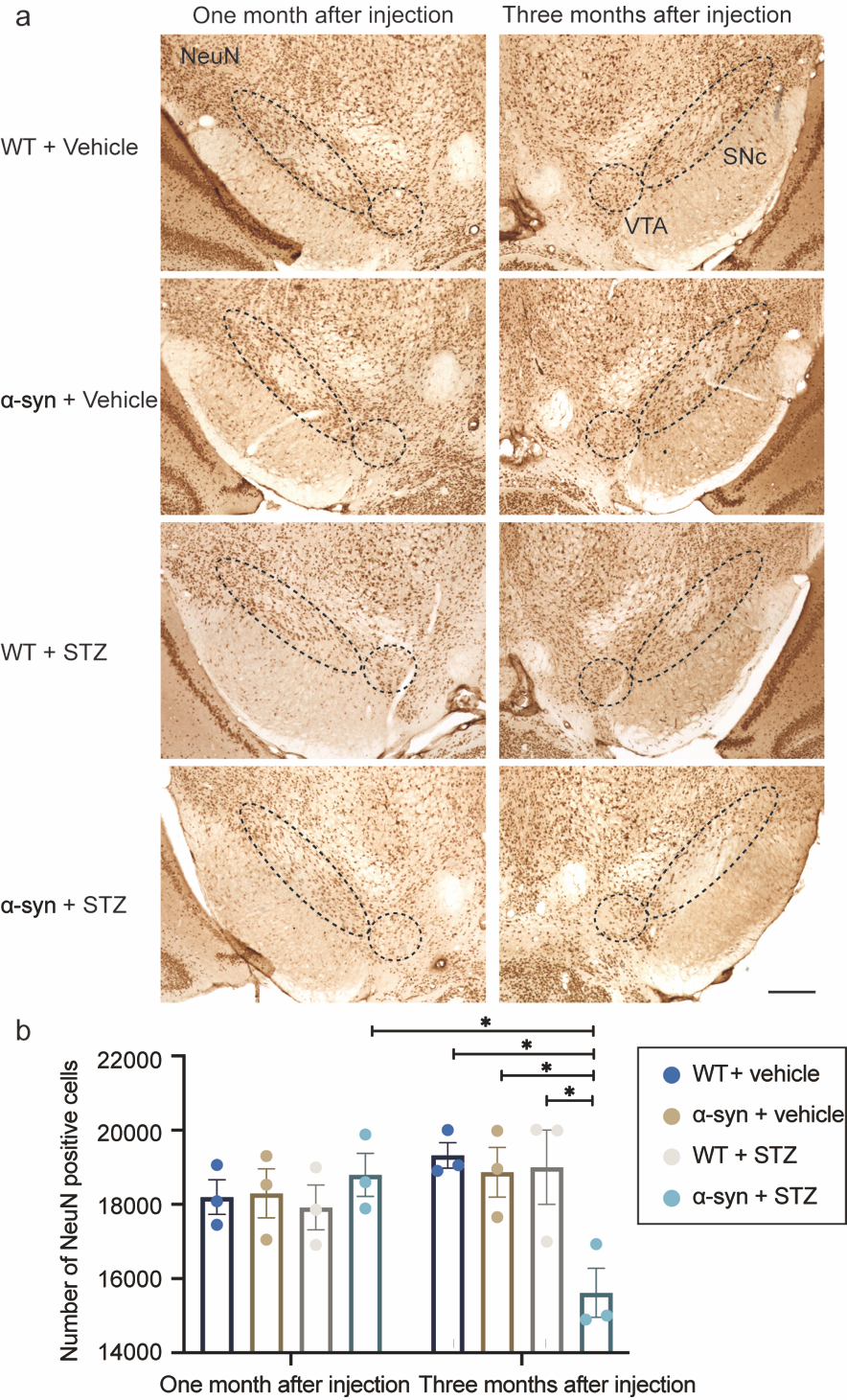


**Fig. S3** (**a**) Representative photomicrographs of coronal mesencephalic sections showing immunohistochemical staining of NeuN in the SN of both WT and BAC-α-syn-GFP mice injected with STZ and vehicle. (**b**) Quantitative analyses of NeuN positive neurons determined by stereology in the SNpc and VTA region. WT + vehicle (n=3/3 mice at 1/3 months after injection, respectively); WT + STZ (n=3/3 mice at 1/3 months after injection, respectively); α-syn + vehicle (n=3/3 mice at 1/3 months after injection, respectively); α-syn + STZ (n=3/3 mice at 1/3 months after injection, respectively). Scale bar: 50 µm. *P<0.05.


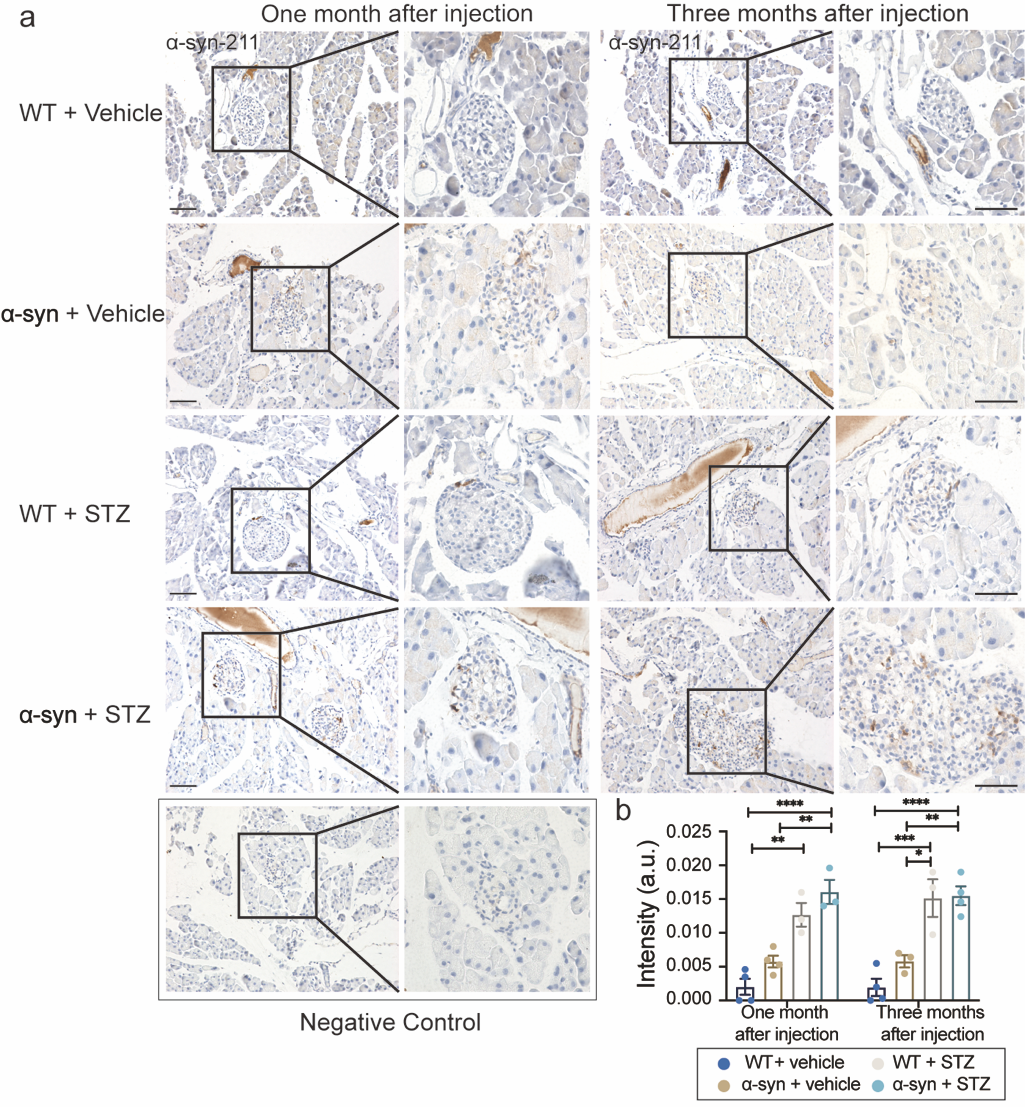


**Fig. S4** (**a**) Representative microscopic images of immunohistochemical staining with antibody specifically against human α-syn at one and three months after injection in the pancreatic islets of WT-vehicle, WT+STZ, α-syn-vehicle and α-syn+STZ groups. (**b**) The semi-quantitative analyses of α-syn intensity (immunohistochemical staining) in the pancreatic islets of WT and BAC-α-syn-GFP mice i.p. injected with STZ and vehicle (n=3 to 4 mice/group). Scale bar: (**a**) left column, 200 µm; right column, 50 µm. *P<0.05, **P<0.01, ***P<0.001, ****P<0.0001.

**
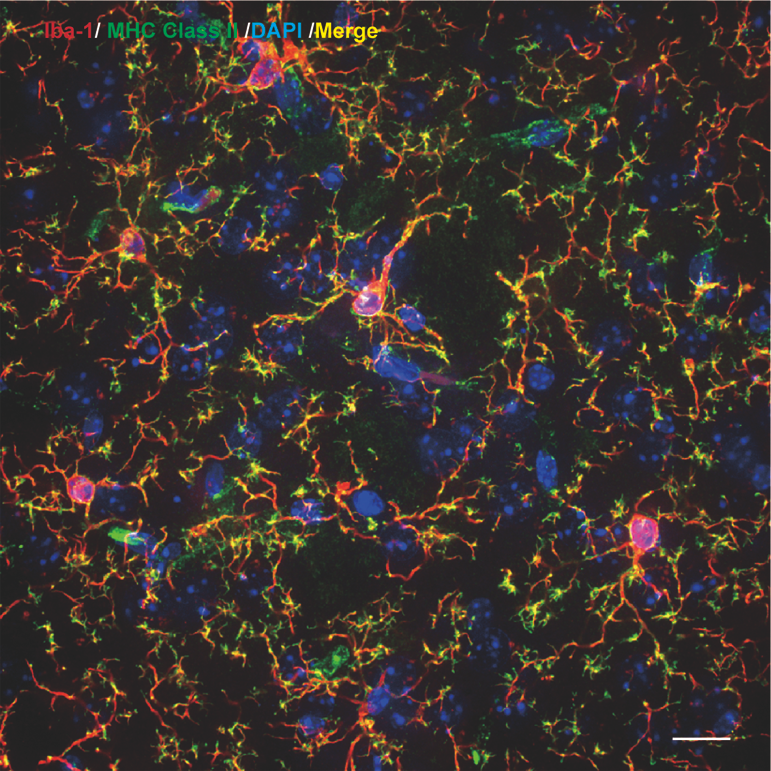
**

**Fig. S5** Dual immunofluorescence for Iba-1 and MHC class II in striatum. Double immunofluorescence labelled with MHC class II (green) and Iba-1(red) antibodies showed co-localization of these two proteins. 3D views of Z-series scanned images from striatum of BAC-α-syn-GFP mice injected with STZ. Image scale bars: 20 µm.

**Table S1** List of the number of animal numbers in each group (total number of mice used in each group, survived number of mice were marked in the brackets).

| **Group** | **One month after injection** | | | |
| --- | --- | --- | --- | --- |
|  | WT + vehicle | WT + STZ | α-syn + vehicle | α-syn + STZ |
| **Number** | 7 (6) | 10 (7) | 8 (8) | 12 (9) |
| **Group** | **Three months after injection** | | | |
|  | WT + vehicle | WT + STZ | α-syn + vehicle | α-syn + STZ |
| **Number** | 8 (8) | 8 (8) | 8 (8) | 12 (7) |

**Table S2** List of the primary and secondary antibodies used for this study and of their working dilutions.

| **Antibody name** | **Catlog** | **Species** | **Source** | **dilution** |
| --- | --- | --- | --- | --- |
| Insulin (C27C9) Rabbit mAb (IHC) | 3014 | Rabbit | Cell Signaling | 1:12800 |
| α-Synuclein (IHC/WB) | Sc-12767 | Mouse | SANTA CRUZ | 1:500/1:1000 |
| Ser-129 phosphorylated α-synuclein (IHC/WB) | ab51253 | Rabbit | Abcam | 1:2000/1:1000 |
| Tyrosine hydroxylase (IHC/WB) | 22941 | Mouse | ImmunoStar | 1:2000/1:1000 |
| Dopamine transporter Polyclonal Antibody (WB) | 22524-1-AP | Rabbit | Proteintech | 1:1000 |
| Anti-Iba1 Rabbit Polyclonal antibody (IHC/IF) | 019-19741 | Rabbit | Wako Chemicals | 1:2000/1:2000 |
| Anti-NeuN antibody(WB/IHC) | SAB4300883 | Rabbit | Millipore | 1:2000/1:1000 |
| Anti-Iba1 antibody (WB) | ab5076 | Goat | Abcam | 1:3000 |
| Anti-GFAP antibody (WB) | ab7260 | Rabbit | Abcam | 1:2000 |
| HLA class I ABC Polyclonal antibody (IF) | 15240-1-AP | Mouse | Proteintech | 1:100 |
| Anti-GAPDH Mouse Monoclonal antibody | 60004-1-Ig | Mouse | Proteintech | 1:2000 |
| Goat Anti-Mouse IgG (H+L), HRP conjugate | ab205719 | Goat | Abcam | 1:1000 |
| Goat Anti-Rabbit IgG (H+L), HRP conjugate | ab205718 | Goat | Abcam | 1:8000 |
| Rabbit Anti-Goat IgG (H+L), HRP conjugate | SA00001-4 | Rabbit | Proteintech | 1:5000 |
| Biotinylated anti-mouse | BA-2000-1.5 | Horse | Vector | 1:500 |
| Biotinylated anti-rabbit | BA-9200-1.5 | Goat | Vector | 1:500 |
| Alexa Fluor® 647-AffiniPure Donkey anti-Mouse IgG (H+L) | 115-605-003 | Donkey | Jackson | 1:500 |
| Alexa Fluor® Cy3-AffiniPure Donkey Anti- Rabbit IgG (H+L) | 711-167-003 | Donkey | Jackson | 1:500 |
